# Birds Decorating Their Nests With Plastic May Suffer Less Egg Depredation by Corvids

**DOI:** 10.1002/ece3.72966

**Published:** 2026-01-19

**Authors:** Tore Slagsvold, Magne Husby

**Affiliations:** ^1^ Department of Biosciences, Centre for Ecological and Evolutionary Synthesis (CEES) University of Oslo Oslo Norway; ^2^ Section of Science Nord University Levanger Norway

**Keywords:** anthropogenic material, corvids, magpies, nest decoration, nest material, nest predation, plastic

## Abstract

Many birds add anthropogenic material to the nest. This may increase the probability of total failure because the nest may be more easily located by enemies. However, the material may also induce a threat response in predators sceptical to new objects (the Neophobia Hypothesis). We presented artificial nests on the ground each with two quail eggs, in territories of Eurasian magpies *Pica pica* in spring. Some nests were decorated with pieces of white plastic while others were not (control). When nests of both types were presented simultaneously on a magpie territory and only a meter apart, depredation started later for nests with plastic than for control nests, supporting the Neophobia Hypothesis. When a trial was repeated on the same territory later in the season, predation started sooner. However, this was probably caused by habituation to the experimental set up (wildlife camera and artificial nests) and not to the plastic itself because in the repeated trials, the eggs in the nests with plastic were still depredated later than the eggs in the control nests. The nests were not depredated sooner if similar experiments had been conducted on the same territory in the previous year. The onset of depredation was no sooner in territories that initially contained plastic close to the magpie nest than in territories containing no plastic. Finally, when only a single nest was presented on a magpie territory, the time lag until depredation was similar for decorated and control nests, suggesting that the increased detectability caused by decoration outweighed the fear response to the plastic. We conclude that the Neophobia Hypothesis may be relevant to natural cases including birds nesting in habitats containing anthropogenic material and to circumstances with repeated visits by corvids to bird nests, such as in a bird colony.

## Graphical abstract

Many birds add anthropogenic material to the nest. We presented artificial nests on the ground in spring, each with two quail eggs and half of them decorated with pieces of white plastic. The plastic induced a neophobic response in territorial European magpies, reducing the rate of nest predation.

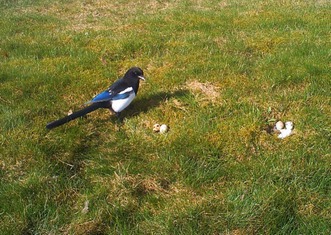

## 1 Introduction

Recently, an increasing number of birds have been found to add anthropogenic material to the nest, such as pieces of plastic (Hansell 2000; Sergio et al. 2011; Jagiello et al. 2019; Sheard et al. 2024; Lambrechts and Deeming 2025). This may increase the risk of total breeding failure because decorated nests may be easier to locate by predators, such as corvids. Corvids are among the most serious predators on bird nests in general (Møller 1987; Madden et al. 2015; Husby and Hoset 2018; Capstick and Madden 2021) and also in our study area in Norway (Husby 2018; Husby and Slagsvold 2025). At the same time, corvids are among the most neophobic species of any bird (Shephard et al. 2015; Greggor et al. 2016). Neophobia is defined as the degree of aversion towards objects or situations that are evolutionarily novel, or the avoidance of an environmental element that has not been experienced previously (Crane and Ferrari 2017). The behaviour is considered to be an adaptive response that increases survival and reproduction (Crane et al. 2020).

The Neophobia Hypothesis proposes that the presence of conspicuous anthropogenic material may cause a threat response in avian predators so that they hesitate to depredate a nest decorated with such material (Husby and Slagsvold 2025). In a recent field experiment presenting quail *Coturnix* spp. eggs in artificial nests on the ground, the hypothesis was supported. Territorial Eurasian magpies *Pica pica* (termed magpies below) seemed to avoid nests decorated with a novel item, namely a shining, metal teaspoon versus a nearby nest with no decoration. Avoidance of such decorated nests also occurred at a landfill where the nests were readily depredated by common ravens *Corvus corax* , although the ravens may have been habituated to such material at the landfill. In both areas, also a third nest was presented near the other two, namely a nest decorated with white hen feathers. Both the magpies and the ravens hesitated to depredate these nests, perhaps because of a fear of being attacked at the site by a bird of prey or by a mammal (Husby and Slagsvold 2025), consistent with the fear of feathers hypothesis (Slagsvold and Wiebe 2021).

The Neophobia Hypothesis will be of little significance if nest predators habituate rapidly to anthropogenic nest material. The alternative and/or additional benefits of using such material may include reduced heat loss of the nest content and/or increased structural support of the nest. Ornamentation of the nest may signal high quality of the builder, which may increase mating success (Borgia et al. 1987) and send a signal of ownership and high social rank to conspecific intruders (Sergio et al. 2011; Mainwaring 2017). Thus, use of anthropogenic material may be considered an extended phenotype (Schaedelin and Taborsky 2009) and not be associated with depredation.

**FIGURE 1:**
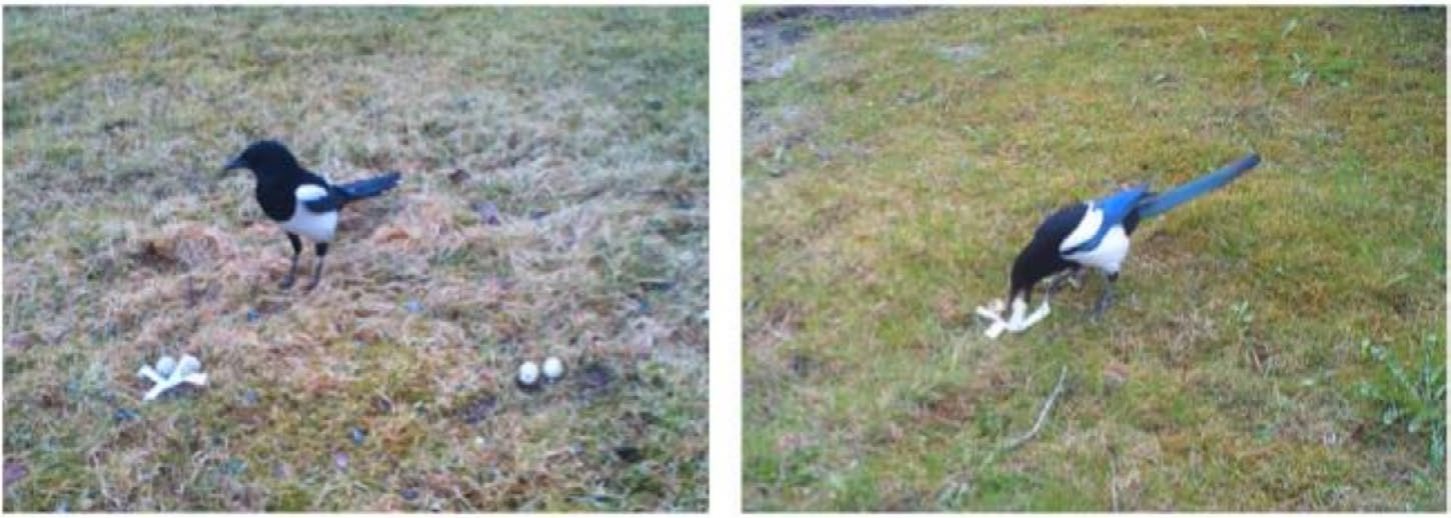
Artificial nests placed on the ground on magpie territories, each containing two quail eggs. Nests were placed 1 m apart in dyads (a: Left), or as single, solitary nests decorated with (b: Right) or without pieces of white plastic. The magpies were able to grasp an egg in their beak (b).

The present study has four objectives. Firstly, we studied whether the nest depredation was related to the ‘natural’ presence of plastic in the respective territories. Secondly, we wanted to repeat the previous experiment on magpies to study whether any fear response exists also when a nest is decorated with plastic and not with a teaspoon or feathers. Thus, we provided territorial birds with a dyad of artificial nests placed on the ground, each containing two quail eggs, where one nest was decorated with pieces of white plastic whereas the other nest was not decorated (control nest; Figure 1a). When both nests were near each other and probably discovered at the same time, we expected from the Neophobia Hypothesis that the magpies would depredate the decorated nest later than the control nest. We also compared the present results with those from the previous trials to study the timing of depredation of decorated nests with plastic, a teaspoon or feathers (using data from Husby and Slagsvold 2025).

Thirdly, we studied whether nests with white plastic were depredated sooner than control nests when only a single nest was provided on each magpie territory (Figure 1b). This might be expected if nests with plastic are discovered sooner than nests without (Sergio et al. 2011). However, the opposite may be expected if neophobia to the plastic is very strong. Finally, we studied how soon the magpies habituated to the presence of anthropogenic nest material by repeating the experiment with white plastic in the same magpie territories after all the eggs had been removed by the birds from the respective nests presented during the first trial.

## 2 Materials and Methods

### 2.1 Study Area and Study Species

The study was done in 2025 with trial sites in gardens in farmland areas located in Stjørdal, Frosta and Levanger communities in Norway (63°26–39′ N, 10°6′‐11′ E). Magpies defend all‐purpose territories (Cramp and Perrins 1994), they are sedentary (Husby 2006), and the adult survival rate is high (Birkhead 1991). Thus, in our study, the focal birds had probably detailed experience within their territory. The magpies start egg‐laying in April and May and care for the offspring until at least August (Husby 1986; Husby and Slagsvold 1992). The mean distance between the successful magpie trial sites was 530 m (SD = 986, range 61–7577 m, *n* = 62). During the first trial on a territory, a minute was spent to observe the presence of plastic on the ground or in trees or bushes, categorised as 1 = no plastic (*n* = 41) and 2 = with plastic (*n* = 21). We only searched a small, central area within 50 m from the respective magpie nest. The habitat was open and the plastic had different colours (white, green, black, blue, transparent) and was easy to detect.

### 2.2 Artificial Nest Experiments

The sample sizes are shown in Table 1. The accepted first trials (*n* = 62) were conducted during 21 March–7 May, and the repeated trials (*n* = 60) during 25 March–12 May. The repeated trials on two territories were not accepted because hooded crows *Corvus cornix* depredated the nests. The artificial nests were always presented in an open habitat with few bushes and trees. They were made by a depression on the ground and contained two quail eggs but no natural nest material (Figure 1), simulating nests of species of ground‐nesting birds in the study area (Husby and Slagsvold 2025). We did three experiments, choosing at random which should be conducted on each magpie territory. In one experiment, two nests were placed only a meter apart to ensure simultaneous detection by the magpies (Figure 1a; termed dyad nests/trials below). By throwing a dice, we decided which nests should be decorated with plastic and which should serve as a control. In the two other experiments, only a single nest was presented on a magpie territory, decorated with plastic or not (Figure 1b; termed solitary nests/trials below). For decorated nests, we used two pieces (2 cm × 13 cm) of white plastic in a cross (Figure 1a,b). We noticed the colour of the plastic rubbish on 21 magpie territories of which 48% were only white and 29% had various colours, including white. The plastic was fixed to the ground by penetration of a 65 mm long galvanised nail so that the pieces should stay in place and not cover the eggs in case of wind. A galvanised nail of the same size was also placed in the middle of every control nest.

**TABLE 1:** (a) Comparison of the time elapsing before quail eggs were removed by European magpies when offered artificial nests on the ground decorated or not with pieces of plastic (see Figures 1 and 2). In 2025, single nests or a dyad of nests were presented on the territories, two trials in each territory. In 2024, a triad of nests were presented once. (b) Egg depredation order in dyad nests in 2025. Data are for first trials and for repeated trials later in the same breeding season and territory. Differences are tested with Mann–Whiteny *U*‐test, or with Wilcoxon paired two sample test.

No	Year	Trials	Nests	Territories	Median (h)	Range (h)	*n*	Test	*z*	*p*
**(a) First egg removed. Time elapsing**										
1	2025	First	Solitary	All	16.6	0.6–255	39			
	2025	First	Dyads	All	3.8	0.9–64	23	*U*‐test	−2.94	0.003
2	2025	Repeated	Solitary	All	6.1	0.2–259	39			
	2025	Repeated	Dyads	All	1.7	0.2–20	21	*U*‐test	−2.38	0.017
3	2025	First	Solitary	No plastic near	10.9	0.6–255	25			
	2025	First	Solitary	With plastic near	19.4	5.6–220	14	*U*‐test	−1.42	0.156
4	2025	First	Dyads	No plastic near	4.2	0.9–64	16			
	2025	First	Dyads	With plastic near	2.9	1.0–10	7	*U*‐test	−0.67	0.504
5	2024	First	Triads	All	6.4	0.1–249	22			
	2025	First	Dyads	All	3.8	0.9–64	23	*U*‐test	−1.75	0.080
6	2024	First	Triads	First egg control	3.3	0.9–64	17			
	2025	First	Dyads	First egg control	6.2	0.1–91	19	*U*‐test	−1.16	0.247
7	2024	First	Triads	Same as in 2025	6.4	0.1–249	22			
	2025	First	Dyads	Same as in 2024	5.2	0.6–220	22	Wilcoxon	−1.12	0.263
8	2025	First	All	Used in 2024	5.2	0.6–220	22			
	2025	First	All	Not used in 2024	7.9	0.9–255	40	*U*‐test	−1.43	0.151
9	2025	First	All	All	7.7	0.6–255	60			
	2025	Repeated	All	All	3.0	0.2–259	60	Wilcoxon	−3.71	< 0.001
10	2025	First	Solitary	Plastic in nest	20.0	0.6–255	20			
	2025	First	Solitary	No plastic in nest	14.8	1.2–127	19	*U*‐test	−0.16	0.877
11	2025	Repeated	Solitary	Plastic in nest	6.2	0.7–259	20			
	2025	Repeated	Solitary	No plastic in nest	4.1	0.2–100	19	*U*‐test	−1.11	0.267
12	2025	First	Dyads	Plastic in nest	4.7	1.0–64	21			
	2025	First	Dyads	No plastic in nest	4.6	0.9–64	21	Wilcoxon	−2.75	0.006
13	2025	Repeated	Dyads	Plastic in nest	1.7	0.1–20	21			
	2025	Repeated	Dyads	No plastic in nest	1.7	0.1–20	21	Wilcoxon	−1.32	0.187
**(b) Egg depredation order**					**Median**	**Rank**				
14	2025	First	Dyads	Plastic in nest	2.57	1–4	46			
	2025	First	Dyads	No plastic in nest	1.47	1–4	46	*U*‐test	−3.44	< 0.001
15	2025	Repeated	Dyads	Plastic in nest	2.56	1–4	42			
	2025	Repeated	Dyads	No plastic in nest	1.39	1–4	42	*U*‐test	−3.18	0.001

We compared the results of a similar experiment conducted partly in the same study area in 2024 where three adjacent, artificial nests with quail eggs were provided on magpie territories (placed in a line of 1 m; data from Husby and Slagsvold 2025; termed triad nests/trials below). In the triad trials, one nest was decorated with a shiny, metal teaspoon (16 g, 13 cm long), one nest was decorated with 10–15 white chicken feathers (3–13 cm long), and one nest was not decorated (control). The quail eggs provided were on average 33.6 mm and 26.5 mm wide and were of a size that enabled the magpies to grasp an egg in the beak (Husby and Slagsvold 2025). Similar quail eggs were used in the experiments in 2025.

In 2025, we followed the same method as adopted by Husby and Slagsvold (2025). The trials always started in daylight (between 07:51–18:47 h of the day, wintertime) and they lasted until all eggs had been removed (after a maximum of 16 days). Behaviours were recorded by a wildlife camera (Wingcam II TL; 8 MP photo resolution), ensuring that the single nests, and both nests of the dyads, were within the field of view. The camera was placed on a 1.5 m high metal pole about 2 m away from the nests. It took a photo every minute independent of the presence of predators and predator activity. In addition, a photo was automatically taken if the predator was detected because of its temperature or movements. We assumed that different magpies were occupying the different trial sites based on their spatial distribution and strong territoriality (see also Section 4).

In 2024, we conducted only a single trial in each territory. In 2025, after a trial had been terminated, we did a second trial of the same type (single nest or a dyad of nests) on the same magpie territory to study habituation. The mean distances between the two trial sites, measured as paced distance, were 11.7 m (SD = 6.0, range 4–28 m). The mean distance of the trial site from the tree with the respective magpie nest was 26.8 m (SD = 28.8, range 1–160 m) for the first trial, and 27.2 m (SD = 28.5, range 2–171 m) for the repeated trial. Before the start of these trials, the respective sites for the two trials within the magpie territory were decided. This was based on guidance from the landowners because most artificial nests were placed in their gardens. Thereafter, we selected an open area away from buildings, trees and bushes but close to the respective magpie nest. Then, two very similar sites were selected, and by throwing a dice, it was decided which of the two sites should be used first. To remember the exact position of the second site, the observer made a sketch of the site but did not put up any mark that could guide the focal magpies to the nest later on. The repeated trial on the same magpie territory started on average 4.8 days (SD = 4.4, range 1–20 days, *n* = 60) after the start of the first trial, depending on the time elapsing before all eggs had been depredated.

### 2.3 Statistical Analysis

We used Apple Preview to analyse the photos, carefully watching every photo during the periods when eggs were removed, to identify the nest predators and to record order and hour of day of egg removals. With the camera in place, the eggs could be seen and thus it was possible to tell whether an egg had disappeared from a nest although the predator was not observed. Quite often the magpies removed the eggs so fast that the detection function of the camera could not take a relevant photo when the predator was present. In all accepted trials, one or more magpie was observed one or more times during a total of 94 of the 122 trials. In a total of 45 trials, a magpie inspected the depredated nest within half an hour after it was empty. If a nest was depredated by a hooded crow (*n* = 4), jackdaw *Corvus monedula* (*n* = 1), dog (*n* = 1) or accidentally destroyed by the landowner (*n* = 1), the trial was excluded.

If an egg had disappeared without any opportunity to notice the predator, we assumed that a magpie was responsible because of the short time interval elapsing between the successive photos and because few nests were depredated by other animals. Likewise, if a magpie was observed removing an egg, or was observed close to a nest during a trial, we assumed that it was a magpie that had removed the other egg also when no predator was directly observed on all the other relevant photos. If a magpie visited the depredated nest within half an hour after the last egg had been removed, we assumed that the nest had been depredated by magpies although no predator was observed during the predation action.

If two eggs were depredated between two successive photos, they were given the same predation order and time. This occurred five times in case of the first egg for the triad trials, and five times for the first dyad trials and five times for the repeated dyad trials. For the solitary nests, this occurred four and one time for nests with plastic, and seven and one time for control nests, in the first and the repeated trial respectively. In all trials, the order of predation was recorded, assigning the same value for each of the eggs depredated simultaneously (e.g., order 1, 2, 2 and 4).

Because magpies normally do not depredate nests during night, we calculated the time elapsing only for the hours between sunrise and sunset for the date in question, using official meteorological data from the study area (https://www.timeanddate.no/astronomi/sol/norge/trondheim). The time elapsing from the start of a trial until the depredation of the first egg did not follow a normal distribution for all variables, nor after log transformation. Therefore, we used non‐parametric tests. For illustration, we used log transformation in Figure 3. SPSS v. 30 was used and two‐tailed tests with a significance level of 0.05.

## 3 Results

### 3.1 Single Versus Dyad Nests

In 2025, the time elapsing before the first egg was depredated on a magpie territory was longer for the trials with single nests (ornamented or not) than for the trials with dyads of nests (Figure 3). This was the case both for the first and the repeated trials on a territory (Table 1, test 1 and 2). Thus, we did separate analyses for the two groups of trials.

### 3.2 Presence of Plastic on the Territories

If a magpie territory contained plastic from other sources than ours near the nest site when the first trial started, we expected the first nest of a dyad to be depredated sooner than when a territory contained no plastic. However, there was no significant difference in time lag between territories with or without plastic near the nest, neither for the first nor for the repeated trials (Table 1, tests 3 and 4). Because of the lack of statistical significance, we did not take the variable into account in the following.

### 3.3 Habituation

In 2024, we presented three artificial nests on each magpie territory, one decorated with a teaspoon, one with white hen feathers, and one without decoration. The times elapsing before the first egg was depredated from any nest during the dyad trials in 2025 were similar to those for the triad trials in 2024 (Table 1, test 5). This was also the case when only comparing the time elapsing for nests where the first egg depredated was from a control nest in both years (Table 1, test 6).

No significant difference was found in the time elapsing before depredation of the first egg during the trials conducted in the same territories in 2024 and in 2025 (Table 1, test 7). Great variation existed in the response between the magpie pairs. If the magpie survival rate was high, we expected a positive relationship between the time elapsing before the first egg was depredated for the same territories in 2024 and 2025. However, no such correlation existed (Spearman rank correlation, *r*_s_ = −0.07, *n* = 22, *p* = 0.753). In 2025, the time elapsing was similar for the first depredated eggs on territories used for the first time and territories also used in 2024 (Table 1, test 8). Therefore, below, we did not consider whether or not the focal territory had been used for trials in 2024.

In 2025, a shorter time elapsed before the first egg was depredated in the second trial compared to the first trial on the same territory (Table 1, test 9). The magpies that spent a long time before depredation started during the first trial were also often late to start depredation during the repeated trial on the same territory (Spearman rank correlation, *r*_s_ = 0.32, *n* = 60, *p* = 0.012). During the first trial, there was no significant correlation between the time elapsing before the first egg was depredated and the time elapsing between the depredation of the first and the final egg. This was the case both for solitary trials and for dyad trials (same type of test; solitary nests: *r*_s_ = 0.13, *n* = 39, *p* = 0.43; dyads: *r*_s_ = 0.07, *n* = 23, *p* = 0.759). Thus, birds that started depredation later did not spend a longer time to depredate all eggs.

### 3.4 Decoration

**FIGURE 2:**
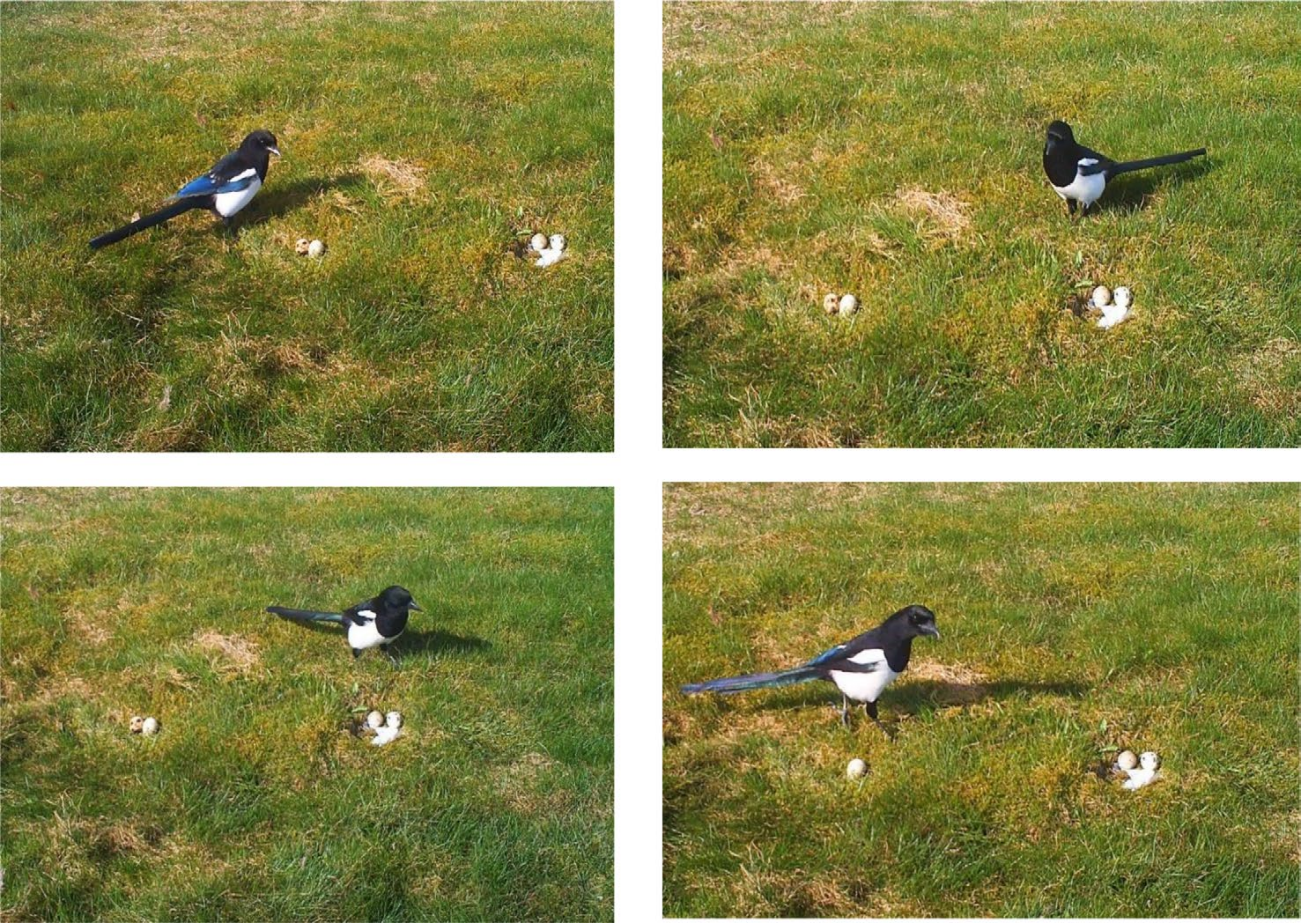
A sequence of photos taken by a wildlife camera showing a trial with two artificial nests each containing two quail eggs, one nest with pieces of white plastic and one control nest. The magpie observed both nests from close range several times (from left to right) before it took an egg from the control nest. The next egg was also taken from the control nest.

When the magpies were offered only one type of nest, the first egg was depredated only insignificantly later in the nests with plastic than in the control nests both during the first and during the repeated trials (Table 1, tests 10 and 11). When the magpies were offered both a decorated nest and a control nest simultaneously on the same territory, the nest with plastic was depredated significantly later during the first trial on the territory but not during the repeated trial although the results were in the same direction (Table 1, test 12 and 13). During both experiments, an egg was taken first from the nest with plastic in only 32% (*n* = 28) of the cases during the first trial, and in only 34% (*n* = 29, eggs taken first) of the cases during the repeated trial on the same territory (note that more than one egg was sometimes judged to be taken first, as explained in the methods). Thus, there were several examples where the magpies inspected both nests at close range on the ground before the control nest was depredated first (Figure 2).

**FIGURE 3:**
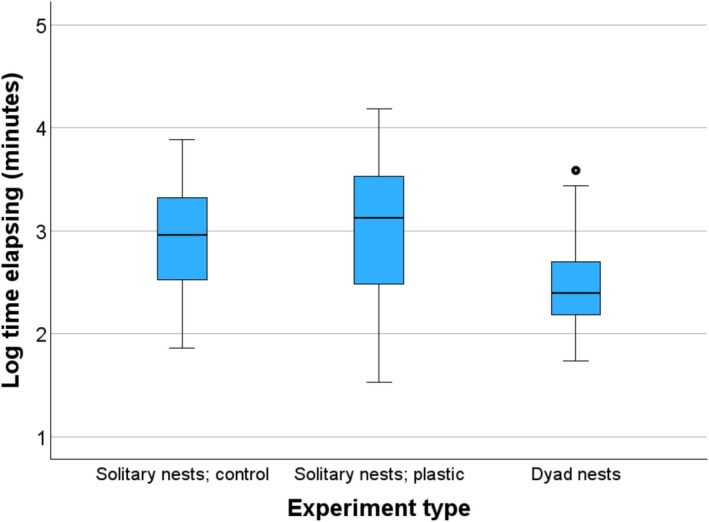
Log_10_ time elapsing from the start of a trial and the time the first quail egg was depredated by a magpie on its territory in trials with solitary control nests, in trials with solitary nests decorated with plastic, and in dyad trials with one nest of each type (see Figures 1 and 2). The box‐plots show the first and the third quartile, the median and the minimum and maximum values. One value outside the whiskers is shown for a dyad trial.

**FIGURE 4:**
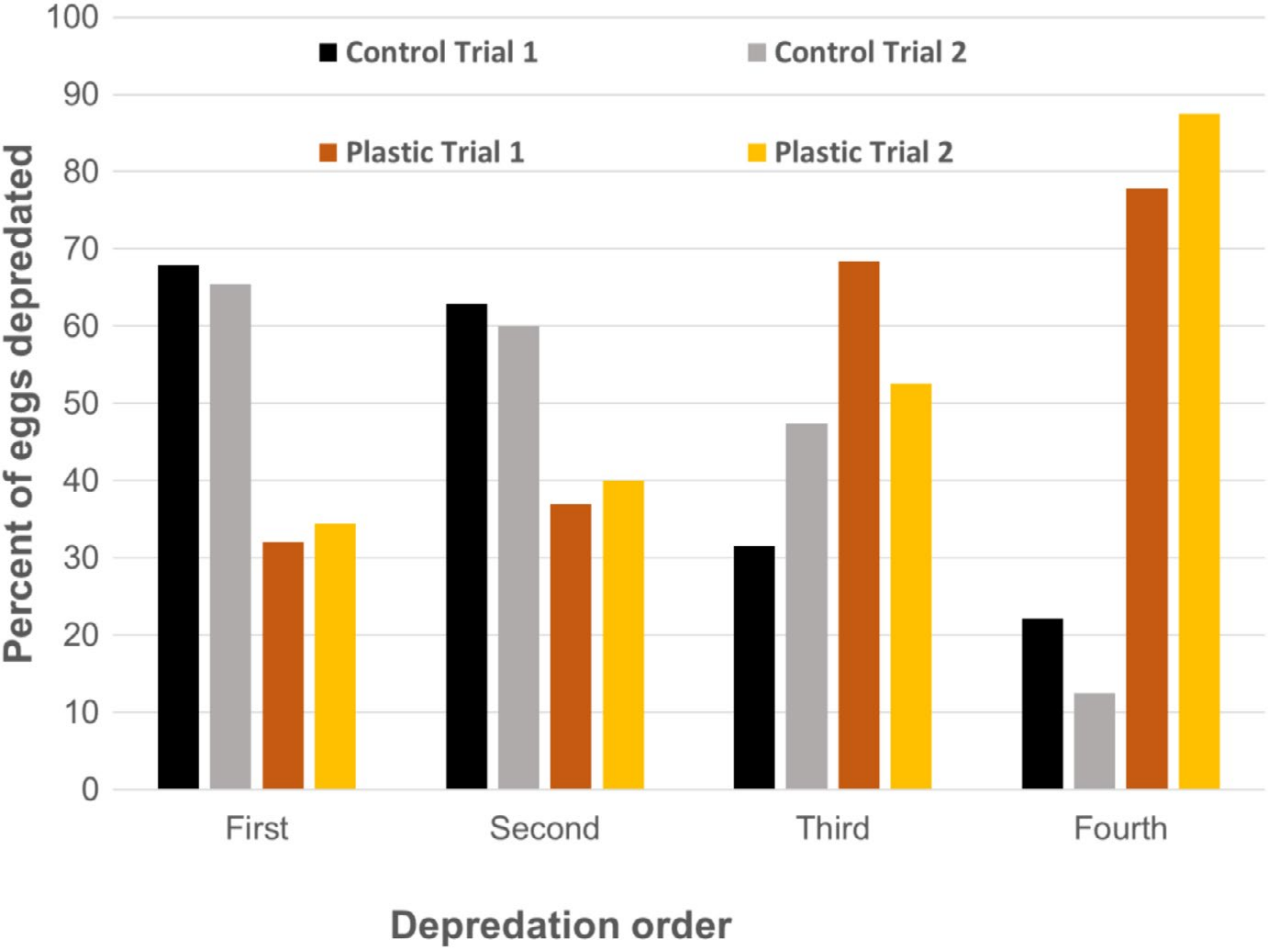
The proportion (%) of the eggs depredated by magpies according to depredation order when a dyad of artificial nests was presented on their territory, each with two quail eggs, and thus with four eggs in total. One nest was decorated with white plastic and one without, serving as a control (see Figures 1a and 2). Trial number (1 or 2) refers to whether the trial was the first on the focal territory, or a trial repeated later in the same season.

During the dyad trials, the order of depredation of the eggs was significantly later in the nests with plastic than in the control nests (Figure 4), both during the first and the repeated trial (Table 1, test 14 and 15). However, within a dyad, the median time interval between the first and fourth depredated egg was short both for the first trials (3.5 min, range 1 min to 62 h, *n* = 23) and the repeated trials (2.3 min, range 0.5 min to 11 h, *n* = 21). This longer time interval between the depredation of the first and the fourth egg in the first trial compared with the repeated trial was statistically significant both for dyads and solitary nests (Wilcoxon paired two sample test; dyads: z = −2.29, *n* = 21, *p* = 0.022; solitary: *z* = −1.987, *n* = 39, *p* = 0.047).

## 4 Discussion

The study has three important findings. Firstly, the initial presence of plastic on a magpie territory did not seem to reduce the fear response of depredating an artificial nest decorated with plastic. Secondly, the time elapsing before the first egg was removed was shorter for the repeated trials than for the first trials on the same territory. However, experience from similar trials in the previous year did not seem to be important. Thus, habituation occurred within but not between seasons. The habituation was probably related to the experimental setup and not to the plastic decoration in the nests themselves, because the order of egg depredation was maintained in the repeated trials. Thirdly, the magpies were more reluctant to start removing eggs from a nest ornamented with plastic than from a nearby nest with no ornamentation, supporting the Neophobia Hypothesis.

### 4.1 Habituation

We studied the effect of habituation in three ways. Firstly, the 'natural' presence of plastic on a magpie territory before a trial started did not reduce the response to the plastic added to the artificial nests. Thus, although in recent years, the amount of plastic is increasing in all sorts of habitats across the world, its presence in a bird nest may still elicit a fear response in corvids. The finding was surprising because European magpies themselves may use plastic as nest material. For instance, in a study in Spain, 95% of the magpie nests had a plastic prevalence (Espinoza et al. 2024). In the present study, plastic was found in 34% (*n* = 62) of the territories within 50 m from the respective magpie nests. We did not study the nest building behaviour of the focal magpies in the present study but in earlier studies (1980–1983, 2013), partly in the same area (2013), no plastic was found in magpie nests (M. Husby, unpublished data).

Secondly, in 2025, we did 40 trials on magpie territories that we had never visited before and 22 on territories used for the triad trials in 2024. If habituation lasted for a year, we expected the depredation in 2025 to start earlier on the latter than on the former territories. This was because the annual survival rate of adult magpies is relatively high. For instance, assuming that the annual survival rate is 70% (Birkhead 1991) and that widowed birds and pairs of birds stay on the same territory if they survive, the probability that at least one magpie of a pair is present in the subsequent year is 91% [= (1–0.3 × 0.3) × 100%]. However, we found no effect of whether or not a previous experiment had been conducted on a magpie territory.

Thirdly, we predicted that the time lag of depredation would be shorter when a trial was repeated later on the same territory in the same season, in particular for the decorated nests because of possible habituation to the plastic. The prediction was supported. However, during the repeated trials, the ornamented nests were still depredated later than the control nests. Therefore, the habituation may have been more a matter of habituation to the experimental setup of a trial, such as to the presence of a video camera on a metal pole, and the presence of quail eggs and artificial nests, than to the ornamentation itself.

Some individual differences were found in the fear response among the magpies consistent between the first and the repeated trials. This may have been due to a difference in the age of the focal pairs, because older, more experienced birds may have been more careful than younger birds. However, as said above, the fear response to ornamentation with plastic was similar when trials were conducted on the same magpie territories in 2025 as in 2024 with presumably many of the same birds present. Furthermore, no significant correlation existed in the fear response for the same territories across the years. However, we did not ring the birds and so we do not know which focal birds had previous experience with the artificial nests and with the quail eggs; neither did we age or sex the focal birds that were active during the experiments. We conclude that habituation occurred within but not between seasons and that individual differences existed among the birds in their reluctance to start depredation within a season.

Wild magpies may recognise individual humans (Lee et al. 2011). However, we did not catch any of the magpies. Thus, the results cannot be explained by disturbance and handling of the birds by humans. We assumed that each bird depredated the experimental nests only on their own territory. The main reason for magpies to visit the territories of other magpies is probably to seek extra‐pair copulations (Buitron 1983; Birkhead 1991). Therefore, male magpies guard their mate carefully during the fertile period (Birkhead 1979). The body size and the conspicuous black and white plumage make a magpie very easy to spot from a distance, causing the intruders to behave cryptically to avoid being attacked (Birkhead 1991). Therefore, it is unlikely that intruders spent time to depredate the artificial nests that always were placed in open habitats. Indeed, we never observed any flocks of magpies; we never recorded more than two magpies simultaneously during the video filming; and on the pictures, we never observed a magpie chasing away another magpie. Our trials were conducted over an extended time period (21 March‐12 May) and thus mostly outside the fertile period of the focal females. Finally, if a magpie had experience from previous depredation of an artificial nest, it would probably show less fear to such nests in a different territory. Thus, our results would be conservative with regard to this potential bias.

### 4.2 Ornamentation

During the dyad trials, the magpies hesitated for a longer period to depredate eggs from the decorated nests than from the control nests. However, it did not take long until also the nests with plastic were depredated. Apparently, after depredation of any nest, the magpies learned that the experimental setup was not dangerous. In turn, this may have increased their appetite to feed also on the eggs provided in the other nests.

If the initial difference in fear was caused by a general fear of novel objects, we expected a difference also to be found when comparing exclusively the decorated and the control nests for the sample of solitary nests. However, this was not found. In case of the solitary nests, we suggest that the fear of novel material was outweighed by the likelihood that the ornamented nests were easier to detect by the predator. Increased detection may also explain why egg predation started sooner in the dyad trials than in the trials with solitary nests. This was somewhat unexpected because all the nests were placed in an open habitat and thus should have been easy to detect (Figure 1). A reward of four eggs from a dyad of nests could increase the risk taking compared to a reward of only two eggs from a solitary nest.

We compared the depredation of eggs for the dyad trials conducted in 2025 with the results for the triad trials conducted in 2024. The time lag was similar, suggesting that the fear of the ornamentation of a nest with white plastic was not different from the fear of a nest ornamented with a teaspoon or with white hen feathers. However, the conclusion should be dealt with some uncertainty because the trials were done in different years, with two nests present in 2025 (1 m apart) and three nests in 2024 (all within one meter), and with the presence of some nest materials during the trials in 2024 (Husby and Slagsvold 2025).

### 4.3 Relevance to Natural Cases

In the present experiments, we used pieces of white plastic which were the most common plastic type in the study area. Such plastic probably helped the magpies to detect the artificial nests and thereby also the quail eggs. It needs to be studied whether the magpies, after nest discovery, also would fear nests that are ornamented with plastic of more cryptic colours. Furthermore, we used artificial nests with no parent bird covering the eggs and the plastic. Thus, experiments should be done where the plastic is added or removed from active bird nests.

In our view, the most important finding of the present study was that for the dyad trials, the order of depredation did not change from the first to the repeated trial on the same territory. Thus, within a bird colony with short distances between open nests, such as in a seabird colony, a corvid may attack a nest without ornamentation sooner than an ornamented nest if the owner is temporarily absent. The predator may soon return to the colony in order to depredate the ornamented nest(s). However, then the nest owner(s) may be attentive and prevent an attack, or the neighbours may be more alert to chase off the predator. If a predator hesitates to depredate a nest with plastic, but stays in the vicinity, as observed with magpies in the present study, it will increase the probability that the returning nest owners and their nesting neighbours will discover the predator. Thus, our results may help to explain why birds add plastic to the nest, in addition to using such material for other purposes, such as nest insulation.

It is also interesting to notice that plastic in the surroundings of the magpie nest did not reduce the scaring effects of plastic inside the nest. Similar benefits from avoiding nest predation may be gained by decorating the nest with other conspicuous objects, such as pieces of metal and/or large, conspicuous feathers. In a study at a landfill, the ravens hesitated to depredate a nest with a shiny, metal teaspoon although such material was common in the local area and was thus experienced by the birds (Husby and Slagsvold 2025).

A second case possibly selecting for decoration of the nest with novel objects may be when the nests of a species are scattered but when each nest is easily discovered by a predator from a distance. A study of the black kite *Milvus migrans* may serve as an example (Sergio et al. 2011; Jagiello et al. 2022). The black kite is a long‐lived, loosely colonial raptor that builds large nests in trees. In the study area in Spain, most kites decorated the nest with white plastic and nests with many such pieces suffered less from usurpation by other kites than nests with few pieces. The authors argued that the plastic served to signal high resource holding potential to aerial receivers, in particular to conspecific intruders.

An additional or alternative explanation to the status signalling hypothesis for the decoration with plastic is the Neophobia Hypothesis, namely that the white plastic elicited a fear response in intruding kites and in avian nest predators. The results may reflect a trade‐off between the cost of increased detectability when decorating the nest with conspicuous, novel material and the benefit of the material of eliciting a fear response in the predator. The cost of increased detectability was illustrated when presenting hen eggs in undefended dummy black kite nests fixed in trees far away (> 500 m) from any active kite nest. The depredation rate was much higher for decorated than for nondecorated nests (depredation rates of 81% and 31%, respectively; Sergio et al. 2011), presumably caused by depredation by corvids and not by mammals because of differences in the foraging behaviour of the two groups of animals. The main cause of breeding failure of kite nests was large mammals (Sergio et al. 2005), a result consistent with the finding that large mammalian predators are not very neophobic (Crane and Ferrari 2017; Husby and Slagsvold 2025). To compare, in the present study, the time lag for nest predation by the magpies was similar for solitary nests with and without plastic. Nest sites of black kites may be used for years and be remembered and revisited by long‐lived predators. In our study, all the artificial nests were placed on the ground in open habitats and probably more readily detected by corvids than the artificial nests used in Spain.

It may be argued that white plastic was present in the surroundings of the kite nests in Spain so that the resident corvids may have been habituated to the material. However, in our study, the presence of plastic close to a magpie nest did not reduce the fear response to the artificial nests, and as mentioned above, ravens at a landfill were reluctant to depredate a nest with a shiny teaspoon although pieces of metal were common in the local area, including some shiny teaspoons (Husby and Slagsvold 2025). Although the plastic may only cause a short‐term fear response in a corvid, it may be sufficient to avoid nest depredation and usurpation until the nest owner returns after a short period of absence.

## 5 Conclusion and Further Studies

The Neophobia Hypothesis suggests that decorating the nest with novel items may reduce nest predation by corvids. Data in support of the hypothesis was found previously when providing territorial magpies and ravens at a landfill with artificial nests with quail eggs on the ground decorated with a shiny metal teaspoon or with large, white hen feathers (Husby and Slagsvold 2025). In the present study, a fear response of resident magpies was also found to nests decorated with white plastic. Surprisingly, habituation to the plastic did not seem to be important. For instance, the fear was no less in territories containing visible plastic near the focal magpie nest than in those with no plastic, and the fear response remained when a trial was repeated later in the same season and territory. Thus, we conclude that the Neophobia Hypothesis may be relevant also to birds nesting in habitats with the presence of anthropogenic material and to circumstances with repeated visits by corvids to bird nests, such as in a bird colony.

We suggest that the delay of nest predation by corvids caused by neophobia may allow parents to return and defend the nest. Further studies are necessary to test this assumption before we can conclude that the delay actually provides an adaptive benefit. In the present study, the magpies chose to take an egg from the nest with plastic first in about one third of the trials. If two thirds of the birds in general hesitate to depredate a nest with plastic, it may have fitness and thus evolutionary consequences. If a significant benefit exists, we may consider an evolutionary arms race to take place, where the predator lags behind the use of new types of anthropogenic material by the nesting birds. However, although magpies are neophobic, they are also quite flexible and opportunistic learners, capable of overcoming initial neophobia. For instance, in the field, but not in captivity, magpies may be reluctant to feed from food provided close to novel, anthropogenic items. Probably, the birds soon become habituated to the anthropogenic material in captivity (Shephard et al. 2015).

It should also be studied whether the magpies have some unknown preferences when foraging that are not part of a neophobic response. Continuos video filming may be useful to compare the time of arrival of the magpies at the setup with the time of actual depredation, and also to study possible startling responses. In the present study, Figure 2 shows an example where a magpie observed both artificial nests from close range several times before it took an egg from the control nest. Finally, we should study the strength of neophobia to anthropogenic material used in bird nests across various species of nest predators, including corvids.

## Author Contributions

**Tore Slagsvold:** conceptualization (equal), data curation (equal), formal analysis (equal), funding acquisition (supporting), investigation (supporting), methodology (equal), project administration (equal), resources (supporting), software (equal), supervision (equal), validation (equal), visualization (supporting), writing – original draft (lead), writing – review and editing (equal). **Magne Husby:** conceptualization (equal), data curation (equal), formal analysis (equal), funding acquisition (lead), investigation (lead), methodology (equal), project administration (equal), resources (equal), software (equal), supervision (equal), validation (equal), visualization (lead), writing – original draft (supporting), writing – review and editing (equal).

## Ethics Statement

The authors have nothing to report.

## Conflicts of Interest

The authors declare no conflicts of interest.

## Supporting information


**Appendix S1:** ece372966‐sup‐0001‐AppendixS1.xlsx.

## Data Availability

The dataset is available in the Appendix S1.
